# Improved Refractive Index-Sensing Performance of Multimode Fano-Resonance-Based Metal-Insulator-Metal Nanostructures

**DOI:** 10.3390/nano11082097

**Published:** 2021-08-18

**Authors:** Yuan-Fong Chou Chau, Chung-Ting Chou Chao, Siti Zubaidah Binti Haji Jumat, Muhammad Raziq Rahimi Kooh, Roshan Thotagamuge, Chee Ming Lim, Hai-Pang Chiang

**Affiliations:** 1 Centre for Advanced Material and Energy Sciences, Universiti Brunei Darussalam, Tungku Link, Gadong BE1410, Brunei; sitizubaidah25@hotmail.com (S.Z.B.H.J.); chernyuan@hotmail.com (M.R.R.K.); roshan.kumara@ubd.edu.bn (R.T.); cheeming.lim@ubd.edu.bn (C.M.L.); 2 Department of Optoelectronics and Materials Technology, National Taiwan Ocean University, Keelung 20224, Taiwan; suyang191@gmail.com

**Keywords:** metal-insulator-metal, refractive index sensor, dual ai stubs, air path, metal defects, finite element method

## Abstract

This work proposed a multiple mode Fano resonance-based refractive index sensor with high sensitivity that is a rarely investigated structure. The designed device consists of a metal–insulator–metal (MIM) waveguide with two rectangular stubs side-coupled with an elliptical resonator embedded with an air path in the resonator and several metal defects set in the bus waveguide. We systematically studied three types of sensor structures employing the finite element method. Results show that the surface plasmon mode’s splitting is affected by the geometry of the sensor. We found that the transmittance dips and peaks can dramatically change by adding the dual air stubs, and the light–matter interaction can effectively enhance by embedding an air path in the resonator and the metal defects in the bus waveguide. The double air stubs and an air path contribute to the cavity plasmon resonance, and the metal defects facilitate the gap plasmon resonance in the proposed plasmonic sensor, resulting in remarkable characteristics compared with those of plasmonic sensors. The high sensitivity of 2600 nm/RIU and 1200 nm/RIU can simultaneously achieve in mode 1 and mode 2 of the proposed type 3 structure, which considerably raises the sensitivity by 216.67% for mode 1 and 133.33% for mode 2 compared to its regular counterpart, i.e., type 2 structure. The designed sensing structure can detect the material’s refractive index in a wide range of gas, liquids, and biomaterials (e.g., hemoglobin concentration).

## 1. Introduction

Recently, refractive index plasmonic sensors based on surface plasmon polaritons (SPPs) [1,2,3,4,5,6,7] have attracted fascinated attention of the experts of biological and chemical detecting since the benefits of nano-size and conquering the diffraction limit [8,9,10,11]. SPPs are arisen from the free electrons on the surface of metals and are firmly confined to metal-dielectric interfaces [12,13]. One such example is a sensor based on the surface plasmon resonance (SPR) [14,15,16,17], a nanometer-sized component that depends on the ultra-high sensitivity of SPPs to the environmental media [18,19]. Therefore, they can be promising candidates to realize highly integrated optical circuits (IOCs) [20,21,22]. Various optical devices have been designed based on SPPs such as filters, directional couplers, modulators, beam splitters, plasmonic diodes, wavelength division multiplexers (WDM), slow light devices, logic gates, all-optical switches, sensors, and so forth [4,23,24,25,26,27,28,29]. Among them, a plasmonic sensor that composes of a metal–insulator–metal (MIM) bus waveguide coupled to a resonator (or several cavities), has become the most promising structure because they acquire the merits of simple form, strong electromagnetic (EM) wave confinement and flexibility, slight band loss, long propagation distance, and ease of fabrication [30,31,32,33,34,35,36,37].

Plasmonic MIM-resonator-based sensors can design through different approaches [38,39,40]. Refractive index-based structures are good candidates for achieving precise and sensitive biosensors. Cavities and different geometrical configurations constructed by the resonators suffer an essential role in engineering a better light–matter interaction in the plasmonic-sensing system. However, as far as the reported articles are considered, the resonator coupling with the MIM bus waveguide is commonly a common circular cavity or a square cavity [41,42,43,44,45]. The best strategy is to choose a circular one. However, if the radius is too large, the devices’ size and full width at half maximum (FWHM) will increase [46]. Light propagation in a rectangular resonator can generate a big angle of the optical path at four right corners and causes significant Ohmic loss [47]. The elliptical-shaped cavity is a better alternative and can enhance the EM field by offering a strong evanescent field, resulting in better light–matter interaction [48,49]. If varying the original resonator can change the equivalent optical path of the Fabry–Perot cavity and influence the SPPs modes in the original resonator, which will excite new optical properties in the plasmonic sensor. Recently, several MIM waveguides with different patterns of resonators have been studied, such as circular/rectangular ring [47], bowtie shaped cavity [50], tooth-shaped cavity [51], X-shaped [52], U-shaped [53], B-shaped [54], T-type [55], M-type [56] and key-shaped [36] resonators, elliptical-shaped trapezoid cavity [57], all-grating racetrack cavity [58], stub coupled with a square cavity [54], metallic nanorods in hexagonal configuration [25], and so forth. 

The elliptical-shaped cavity is one of the most effective resonator types in plasmonic sensors because of their simplicity in the design/manufacture process and tunable resonance wavelengths. Besides, they have a better coupling capability compared to the circular-disk resonators. As a result, different plasmonic sensors could be designed based on such resonators. For example, in [48], the authors employed an elliptical resonator with a silicon strip layer to obtain a high Q-factor and a sensitivity of 550 nm/RIU. In [59], the authors used a half-elliptical groove and an elliptical cavity resonator and achieved the transmission and group index of 90% and 63. On the other hand, Zafar et al. [60] proposed a sensor based on the double elliptical ring resonators with an 1100 nm/RIU-sensing sensitivity. Accordingly, in [49], elliptical-shaped resonators with two rectangular stubs coupled to MIM bus waveguide were used to obtain both Lorentzian resonance mode and Fano resonance mode. In addition, Salah E et al. [61] designed a structure comprising an elliptical-like racetrack cavity, resulting in a refractive index sensitivity of 1400 nm/RIU.

Furthermore, in [36], the authors proposed an elliptical-like structure coupled with a key-shaped resonant cavity, which yielded 1261 nm/RIU sensitivity. However, most current systems produce only one Fano resonance mode and cannot simultaneously achieve multi-tuned Fano resonance modes, high sensitivity, and an acceptable figure of merit (FOM). Using stub-shaped resonators is another technique, which could apply to design optical sensors based on cavity plasmon resonance (CPR) [46,62,63,64]. Another resonator pattern used metal nanorods or baffles to enhance the gap plasmon resonance (GPR) [25,65]. In addition, an air path formed in a resonator can induce more resonance mode due to the increase of dipolar effect along the air path [66]. Our designed sensor has the potential to change this. Based on previous literature, incorporating cavity regions and metal defects can change the resonance modes and enhance the coupling efficiency in a plasmonic system. Therefore, we innovatively created dual air stubs, Ag nanorods, and an air path on the built-in metal component to improve the sensor performance.

Among the various sensors, the surface plasmon Fano resonance-based waveguide system has become a favorable alternative in sensing applications. Fano resonance is a quantum interference phenomenon that results from the destructive interference between a broad continuum state and a narrow discrete one [30,32,42,44,45,63,67,68], which is entirely distinct from conventional Lorentz resonance. Fano resonance mode exhibits asymmetric and sharp spectral line profile and substantial EM wave enhancements. These features show potential applications in photonics devices. This paper designs a plasmonic sensor structure composed of a MIM bus waveguide, including several metal defects and dual air stubs side coupled to an elliptical-shaped resonator containing an air path. This kind of waveguide-based sensor has an inherent advantage to achieve high IOCs.

Furthermore, the double air stubs and an elliptical air ring can behave as a Fabry–Perot cavity, facilitating the Fano resonance modes. Our simulation results indicate that the proposed structure can offer an ultrasharp and asymmetrical Fano resonance mode in the transmittance spectrum. When TM incident EM wave impinges the MIM bus waveguide, the SPPs excited on the metal surface and coupled to the elliptical-shaped resonator through the air optical path, thus originating sharp asymmetry Fano resonance in the proposed plasmonic-sensing system. The numerical simulation can perform by using the finite element method (FEM) to investigate the influence of structural parameters and environmental media on the transmittance spectrum, which realizes the tuning of multiple Fano resonance modes. By optimizing these structural parameters, the proposed type 3 structure can obtain high sensitivity and an acceptable figure of merit. 

We organized this work as follows: Section 2 introduces the basic sensor structure used to design the initial plasmonic sensor system. Besides, the simulation method and basic formulas are involved. Section 3 proposes type 1 and type 2 plasmonic sensors and demonstrates two and five transmittance dips, respectively. Section 4 proposes the type 3 structure by adding dual air stubs and Ag nanorods based on the type 2 structure. Besides, we summarize the results and compare them with some recently published literature. In Section 5, we apply the type 3 structure for the detection of hemoglobin concentration. Finally, the last section is devoted to conclusions.

## 2. Structure Design and Simulation Method

**Figure 1:**
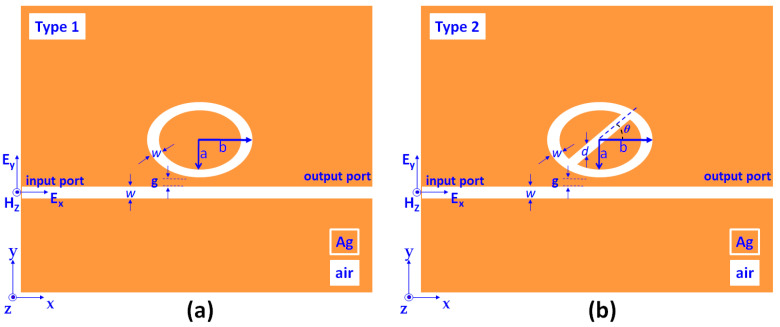
Schematic diagrams and geometric parameters of the proposed (**a**) type 1, and (**b**) type 2 plasmonic MIM-cavity sensor system.

First, two plasmonic sensor structures are investigated and compared, i.e., the plasmonic MIM bus waveguide side-coupled with an elliptical ring cavity (termed the type 1) and an air path embedded in type 1 (termed as type 2). Figure 1a,b shows the top view of the type 1 and type 2 MIM-cavity configurations involving a bus waveguide (width *w*) and an elliptical air ring (width *w*) with an air path (width *d*). In Figure 1b, the air path’s direction can be defined by an angle of *θ* between the *x*-axis and the center of the air path. The semiminor and semimajor axes of the inner ellipse are *a* and *b*, and the semiminor and semimajor axes of the outer ellipse are *a* + w and *b* + w, respectively. The coupling distance between the bus waveguide and the elliptical-shaped ring resonator is *g*. The proposed structure is a two-dimensional (2-D) model, and the golden and white parts in Figure 1a,b denote silver (Ag) and air (*n* = 1), respectively. In the simulations, the structure in the *z*-direction can be considered as infinite. The insulator material used to fill the systems is air. Meanwhile, the metal material of the substrate area is assumed to be silver, which can be obtained from the Drude model [69].

(1) $$\varepsilon_{\mathrm{Ag}}\left(\omega \right)=\varepsilon_{\infty }-\frac{\omega_{p}^{2}}{\omega^{2}+i\omega \gamma }$$

where ε_∞_ = 3.7 stands for the infinite dielectric constant, *ω* = 9.10 eV is the frequency of the incident light, ω_p_ denotes the bulk plasma frequency, and *γ* = 18 meV represents the electron collision frequency. The elliptical ring and an air path in the resonator can function as a Fabry–Pérot cavity. The SPPs can arouse when the incident EM wave approaches near the intrinsic resonant wavelength (λ_res_) for a MIM waveguide-coupled resonator. The congregated phase shift per cycle travel for the SPPs in the resonator is

∆φ = 4π × Re(*n*_eff_) × *L_eff_*/λ + 2φ (2)

where *n*_eff_, φ, and *L_eff_* are the effective refractive index of the SPPs, the phase shift arises from the SPP reflection off the metal wall in the resonator, and the effective lengths of the cavity, respectively. Constructive interference will appear if ∆φ = 2mπ (m is the resonant order, m = 1,2,3,…) and thus the

λ_res_ = 2n_eff_*L*_eff_/(m − φ/π) (3)

Only that incident wave’s wavelength content with Equation (2) can travel effectively, while it will impede others. From Equation (3), the λ_res_ is proportional to *L*_eff_ and *n*_eff_, and the proportionality coefficient is

dλ_res_/d*L* = 2*n*_eff_/(*m* − φ/π) (4)

where *n*_eff_ can be describe as:

(5) $$n_{\mathrm{eff}}={\left(\varepsilon_{\mathrm{Ag}}{}^{}+{\left(\frac{k_{}}{k_{0}}\right)}^{2}\right)}^{1/2}$$

where, k = 2π/λ is the wave vector in the waveguide and k_0_ is the wave vector in the free space. 

Simulations were performed by a 2-D FEM (COMSOL Multiphysics) with perfectly matched layer (PML) absorbing boundary conditions for absorbing departing EM waves at the simulation’s boundaries. The transmittance (*T*) can be calculated by the ratio of output power (*P*_out_ = ∫*P*_oavx_dS_2_) to the input power (*P*_in_ = ∫*P*_oavx_dS_1_), i.e., *T* = *P*_out_/*P*_in_, where *P*_oavx_ is the component of time-averaged power in the *x*-axis. To excite a surface plasmon, we need a component of E field acting along the surface in the same direction as the wave vector. TM is normal/perpendicular to the boundary between metal and dielectric, and SPR only can be induced under TM polarization state. In a practical situation, the TM-polarized incident wave can pass the input port of the bus waveguide and be measured at the output port [70,71,72]. We use *S* = Δλ/Δ*n* (nanometer per refractive index, nm/RIU) to calculate the sensitivity (S), where Δλ is the λ_res_ shift of transmittance, and Δ*n* is the difference in the refractive index corresponding to λ_res_. Full-width half-maximum (FWHM) can be defined as the bandwidth value connected to the left and right of the half-high position of the λ_res_ dip/peak in the transmittance spectrum. The influence of the bandwidth of localized SPR on the sensitivity can be expressed by the figure of merit. There are two definitions for calculating figure of merit, FOM and FOM* [61,73,74], i.e., FOM = S/FWHM, and FOM* = ΔT/TΔn, where T denotes the transmittance, and ΔT/Δn is the transmission change at a fixed wavelength induced by a refractive index change. We used the FOM because we clarify the optical properties based on FWHM. Besides, Q factor can be obtained by λ_res_/FWHM [75].

## 3. Investigation of Sensor Structure with Transmittance Dips in Type 1 and Type 2 Structures

**Figure 2:**
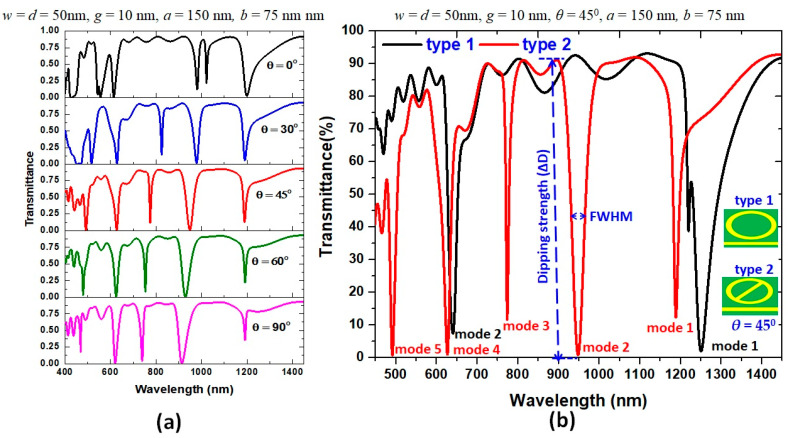
(**a**) Transmittance spectrum as a function of *θ* between the directions of air path in inner ellipse and *x*-axis. (**b**) Transmittance spectra of type 1 and type 2 structures at *θ* = 45° in the wavelength range of 450–1450 nm. The structural parameters are on the top of the figures.

Based on Equation (3), λ_res_ is proportional to the structural size and effective refractive index, *n*_eff_. Therefore, the coupling angle of EM wave can mediate the interaction between the bus waveguide and resonator and significantly influence the transmittance spectrum’s line shape. For simplicity, except *θ*, we do not debate the optimization of geometrical parameters on the plasmonic responses in type 1 and type 2 structures, but directly provide the optimized value, w = 50 nm, *g* = 10 nm, *d* = 50 nm, *a* = 150 nm, and *b* = 75 nm, respectively. Besides, we define the difference between the maximum and minimum transmittance as the dipping strength (i.e., ΔD = T_max_ − T_min_), as shown in the inset of Figure 2b. First, we inspect the influence of *θ* on the transmittance spectrum. Figure 2a shows the transmittance spectrum as a function of *θ* when the other structural parameters are on the top of this figure. In Figure 2a, we found that *θ =* 45° is an optimal angle for effectively coupling the bus waveguide to resonator owing to a deeper ΔD and a narrower FWHM compared to other values of *θ*. Thus, we use *θ* = 45° for the successive simulations. Figure 2b examines the transmittance spectrum of the resonance modes for type 1 and type 2 structures in the wavelength range of 450–1450 nm. We illustrated the structural parameters on the top of this figure. Due to the destructive interference between the narrow discrete state of the resonator and the broad continuum state of the bus waveguide, Fano resonance modes were excited. The role of type 1 functions as a reference structure for the designed sensing system. In Figure 2b, we can find only two transmittance dips corresponding to Fano resonance mode at λ_res_ = 1251 nm (mode 1) and λ_res_ = 641 nm (mode 2) in type 1 structure. In type 2 structure, we observed multiple Fano resonance modes corresponding to five transmittance dips occurring at λ_res_ = 1189 nm (mode 1), 949 nm (mode 2), 774 nm (mode 3), 627 nm (mode 4), and 492 nm (mode 5), respectively. In addition, the ∆D at the λ_res_ of modes 2, 4, and 5 in type 2 structure is lower than 1%, indicating the type 2 structure behaves as a better light–matter coupling between the bus waveguide and resonator than that of type 1 structure. When an air path appears in the resonator, the coupling of two discrete narrowband states (i.e., an elliptical ring and an air path) and a broad continuous form (i.e., bus waveguide) will induce five Fano resonance modes in the plasmonic system, as is shown in the red curve in Figure 2. Compared to the two resonance modes found in the type 1 structure, the resulting five Fano resonance modes in the type 2 structure can attribute to the resonator’s symmetry breaking, arising from the inherent resonant mode in the air path. Table 1 shows the λ_res_, FWHM, ΔD and Q-factor of type 1 and type 2 structures at corresponding resonance modes. It indicates that the SPR and CPR effects in type 2 structure would increase due to the introduction of the air path. This information offers a valuable insight into the function of the air path in the type 2 structure.

**Figure 3:**
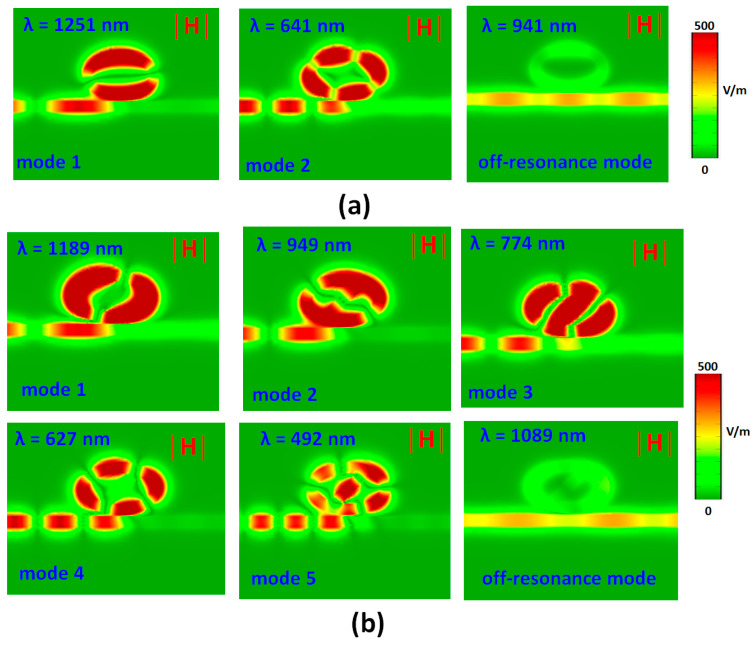
Truncate views of normalized magnetic field (|H|) intensity of (**a**) type 1 and (**b**) type 2 at corresponding resonance modes and one of the off-resonance modes, respectively.

When the incident EM wave’s wavelength contents with the resonance condition in the MIM-cavity system, the SPPs’ energy can transfer from the bus waveguide to the resonator through near-field coupling and permits the construction of a stable standing wave mode in the resonator, which can effectively modulate the transmittance spectrum of the plasmonic system. To further understand the physical nature of resonance modes that occurred in the investigated system, Figure 3a,b compares the normalized magnetic fields (|H|) of type 1 (Figure 3a) and type 2 (Figure 3b) structures at corresponding resonance mode (i.e., at transmittance dip) and off-resonance mode (i.e., at high transmittance), respectively. It is evident from Figure 3a,b that the SPPs wave can well couple to the elliptical ring resonator through the air path at λ_res_, which can create the standing-wave field patterns between the bus waveguide and the resonator. However, SPPs are restricted predominantly to the left side of the bus waveguide and the resonator. As a result, SPPs cannot propagate on the right side of the bus waveguide, which is consistent with the observation of low transmittance (i.e., long ΔD) at λ_res_. The incident wave’s wavelength influences the |H| field distributions at corresponding λ_res_ with different phases. Besides, the dipole effect, i.e., positive-negative charge pairs, may induce along the two sides of the air path. This observation hints that the CPR caused by the air path can significantly contribute to the field enhancement in the proposed type 2 structure. The air path embedded in the elliptical-shaped resonator allows the mighty confinement of SPPs and provides destructive interference in the resonator. Thus, the apparent transmittance dips could be achieved in Figure 2a,b, which impedes energy transmission. When λ_res_ remains at the off-resonance mode, the SPPs wave hardly remains at the resonator, revealing that the SPPs wave in the bus waveguide has a constructive interference. The |H| field distributions exhibit standing wave-like patterns on the metal surface with a remarkable field enhancement since the hybridization of SPR and CPR [17,76,77,78]. 

**Figure 4:**
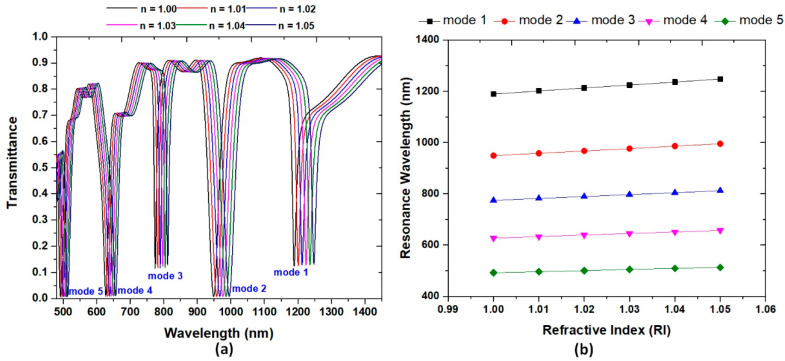
(**a**) Transmittance spectrum of the type 2 structure from mode 1 to mode 5 when the surrounding refractive index, *n*, varies from 1.00 to 1.05 with the interval of 0.01, respectively. (**b**) Calculated resonance wavelength (λ_res_) of type 2 structure from mode 1 to mode 5 versus the refractive index, *n*, in the range of 1.00–1.05 with the interval of 0.01. The structural parameters are the same as those used in Figure 2b.

When the type 2 structure involves the detecting medium, the *n*_eff_ in the bus waveguide and resonator is varied and is remarkably responsive to the ambient material. Figure 4a displays the transmittance spectrum of type 2 structure with the varying refractive index, *n*, in the range of 1.00 to 1.05 with the interval of 0.01. The other structural parameters are the same as used in Figure 2b. As observed in Figure 4a, the transmission peak redshifts with the increase of *n* because of the increase of *n*_eff_, exhibiting a little refractive index change (∆*n*) and resulting in a remarkable λ_res_ shift. The five resonance dips all have a linear relationship with *n,* which agrees with Equation (3). Figure 4b shows the calculated λ_res_ of type 2 structure from mode 1 to mode 5 versus the refractive index, *n*, in the range of 1.00–1.05 with the interval of 0.01. The obtained sensitivity and FOM are 1200 nm/RIU and 80.00 1/RIU for mode 1, 900 nm/RIU and 31.14 1/RIU for mode 2, 800 nm/RIU and 80.00 1/RIU for mode 3, 600 nm/RIU and 40.00 1/RIU for mode 4, and 400 nm/RIU and 40.00 1/RIU for mode 5, respectively. These values can satisfy the commercial application of refractive index sensors.

## 4. Investigation of Sensor Structure with Transmittance Peaks in Type 3 Structure

**Figure 5:**
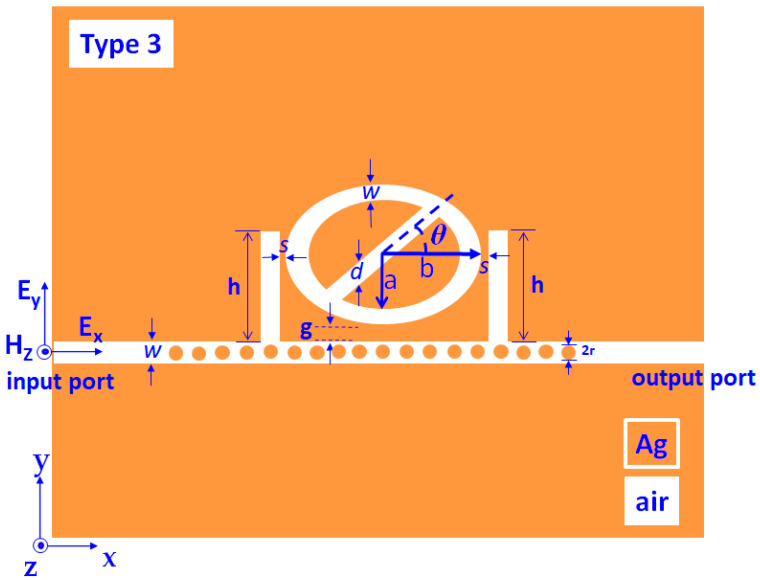
Schematic diagram and geometric parameters of the proposed type 3 structure.

Other combinations of air stubs and metal defects can introduce in the type 2 structure to improve the sensor performance and functionality of the plasmonic-sensing structure. Figure 5 shows the schematic diagram of type 3 structure which is modified based on type 2 structure, comprising dual air stubs (with a width of *w*, the height of *h*, and gap distance of *s* between the stubs and the resonator), and *N* number of Ag nanorods (with the radius of *r* nm) uniformly distributed in the bus waveguide. The resonance condition of SPP waves, including SPR, CPR, and GPR modes, is changed in the type 3 structure due to the interference of EM waves among the air stubs, air gaps among adjacent Ag nanorods, metal surfaces, and the air path in the elliptical-shaped resonator. 

**Figure 6:**
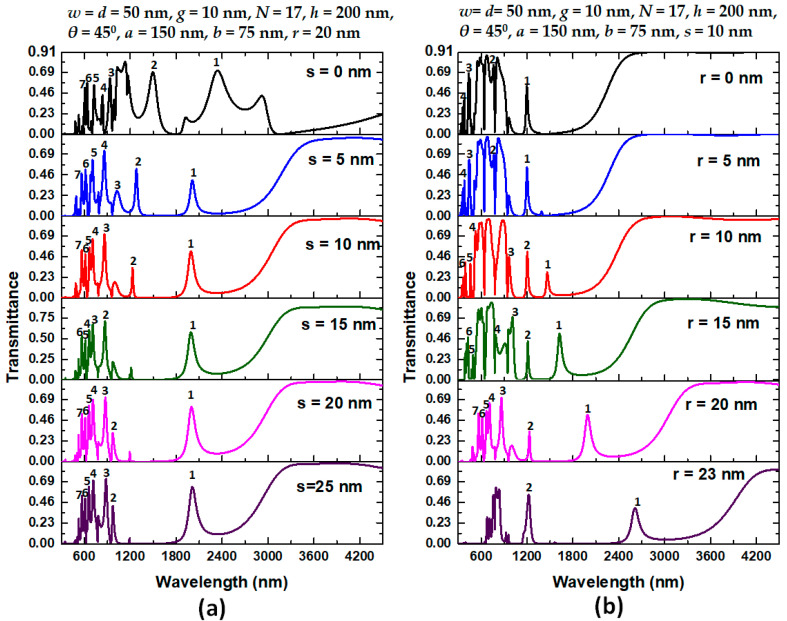
(**a**) Transmittance spectrum of the proposed type 3 structure for varying coupling distance (*s*) between stubs and elliptical-shaped resonator from 0 to 25 nm in the step of 5 nm and (**b**) for varying Ag nanorod’s radius (*r*) in the range of [0, 5, 10, 15, 20, 23] nm, respectively. The other structural parameters are on the top of the figures. We labeled the available resonance modes in the figures.

Subsequently, we inspect the influence of structural parameters on sensitivity and FOM of the proposed type 3 structure and investigate four key influencing structural parameters, *s*, *r*, *N*, and *h*, in our design. Figure 6a,b shows the transmittance spectrum of the proposed type 3 structure for varying the coupling distance (*s*) between the air stubs and elliptical-shaped resonator from 0 to 25 nm with an interval of 5 nm and for changing the Ag nanorod’s radius (*r*) ranging in [0, 5, 10, 15, 20, 23] nm, respectively. The other structural parameters are shown on the top of the figures. We labeled the available Fano resonance modes in the figures based on transmittance value, line shape, and FWHM. As seen in Figure 6a,b, the transmittance curves have different pictures of changes in structural parameters since their distinct physical mechanisms. They reveal other mode numbers concerning different structural parameters and resonance conditions. Note that an entirely different profile of transmittance spectrum compared to type 2 structure is achieved. We found that the transmittance dips in type 2 structure (see Figure 2) are dramatically changed into transmittance peaks and formed multiple Fano resonance modes (Figure 6a,b), when the stubs and the metal defects can be included in the plasmonic system. The generated multiple Fano resonance modes have instinctive merit to achieve high sensitivity. The type 3 structure can provide flexible control over the resonance modes compared to its regular counterpart, i.e., type 1 and type 2 designs. This point gives valuable insight into the function of the stub and Ag nanorods on/in the bus waveguide. The coupling distance *s* associates with coupling effects and influences the interaction between the stubs and resonator. As observed in Figure 6a, the transmittance peaks blueshifts with the increasing *s*, and the coupling effect closely relates to *s* and turns weaker because of the increase of *s*. The resonator is strongly affected when the distance between stubs and resonator is zero (i.e., *s* = 0). Different transmittance value means the additional propagation loss. The Ag nanorods set in the bus waveguide can serve as a buffer to mediate the GPR mode, improving the bus waveguide and resonator coupling effect.

As indicated in Figure 6b, the changing *r* results in redshifts with the increasing of *r*. Note that the variation of *r* in mode 1 significantly influences shifting λ_res_ from 1209 nm to 2639 nm, showing an apparent redshift of 1428 nm as *r* increases from 0 nm to 23 nm. Therefore, we can tune the Fano resonance peak to the anticipated wavelengths by changing the radius r. As seen, only four modes are available when *r* = 0 nm (i.e., no metal rods) and *r* = 5 nm, and two workable modes when *r* = 23 nm. Therefore, the optimal range of *r* is in the field of *r* = 15 nm to 20 nm. If the *r*’s value is too small, it becomes difficult to fabricate the device; yet, if the *r*’s value is big, the FWHM is large, which is not meaningful. After careful comparison in Figure 6a,b, we can choose the optimal values of *s* and *r* as *s* = 10 nm and *r* = 20 nm based on the profile of Fano resonance modes and FWHM. 

**Figure 7:**
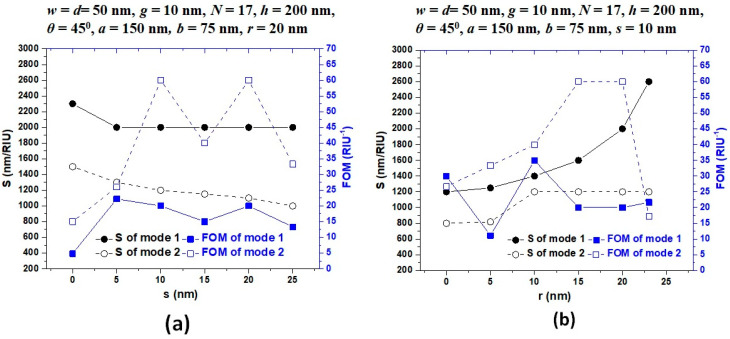
Sensitivity and figure of merit (FOM) of the proposed type 3 structure in mode 1 and mode 2 for varying (**a**) coupling distance between stubs and resonator (s) from 0 to 25 nm in the step of 5 nm, (**b**) varying Ag nanorod’s radius (*r*) in the range of [0, 5, 10, 15, 20, 23] nm. The other structural parameters are on the top of the figures. The surrounding refractive index varies from 1.00 to 1.05 with an increment of 0.01.

Figure 7a,b depicts the sensitivity (nm/RIU) and figure of merit (1/RIU) of mode 1 and mode 2 for the variation of *s* and *r* in type 3 structure. For simplicity, we only discussed the sensitivity (S) performance of mode 1 and mode 2, because the S values obtained from mode 1 and mode 2 are much higher than those of other modes. It is evident in Figure 7a that the structure’s sensitivity can get above 2000.00 nm/RIU when the value of *s* varies in the range of 0–25 nm, while the structure’s sensitivity can increase from 1200 nm/RIU to 2600.00 nm/RIU as the value of *r* varies from 0–23 nm in mode 1, showing the robustness of fabrication. It is worth noting that with the increasing *r*, the sensitivity shows a linear growth trend. Therefore, the higher the *r*, the greater the sensitivity. Thus, the *r* is a favorable factor for the sensing performance in the type 3 structure. Besides, the FOM values are acceptable for the application in commercial sensors. Based on the results obtained from Figure 6 and Figure 7, *s* = 10 and *r* = 20 nm can be regarded as the typical values in the subsequent optimization process.

**Figure 8:**
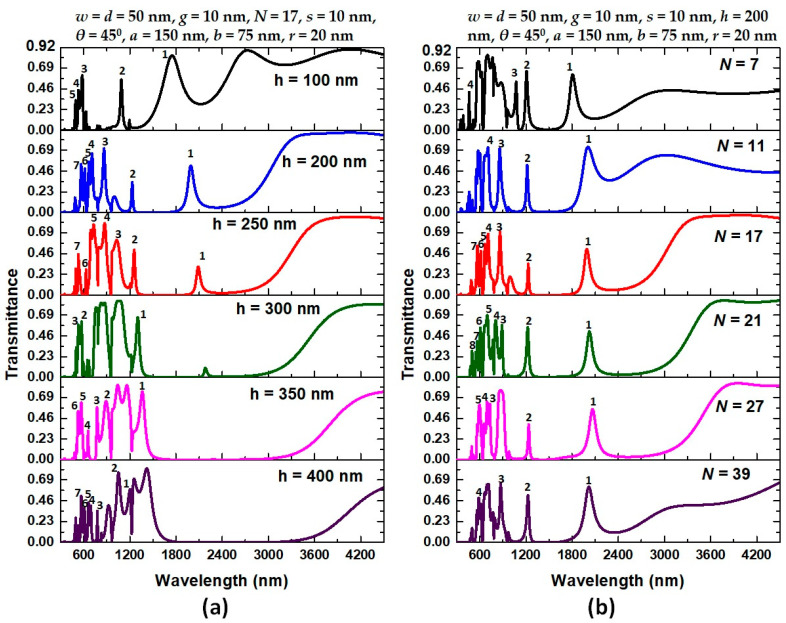
(**a**) Transmittance spectrum of the proposed type 3 structure for varying the stub’s height in the range of [100, 200, 300, 350, 400, 450] nm and (**b**) for varying the number of Ag nanorod’s radius (*N*) in the range of [7, 11, 17, 21, 27, 39] nm, respectively. The other structural parameters are on the top of the figures.

Based on the above results, we note that the sensing performance can improve by three factors, i.e., (1) breaking resonator symmetry by an air path, (2) enhancing GPR effect in the bus waveguide by metal defects, and (3) increasing coupling effect between bus waveguide and resonator by dual air stubs. These findings revealed that adjusting the *h* and *N* on the type 3 structure is also an efficient method for tuning the Fano resonance. Next, we examine the influence of the height of dual air stubs (*h*) and the number of metal defects (*N*) on the transmittance spectrum of type 3 structure, as shown in Figure 8a,b, respectively. The structural parameters are on the top of the figures. It can note from Figure 8a that as the value of *h* increases from 100 nm to 300 nm, the overall transmittance spectrum tends to shift and decrease to longer wavelengths, and this trend vanishes when *h* changes from 350 nm to 450 nm. Thus, the Fano resonance peaks will significantly reduce with the increase of *h* due to the more negligible coupling effect of a larger *h*. We can understand this phenomenon because of a suitable height of dual air stubs that can give a better light coupling between the air stubs and resonator. Thus, an efficient CPR effect formed in the air stubs can determine an appropriate value of *h*. As observed, the optimal value is *h* = 300 nm, which can support more Fano resonance modes ranging from visible to infrared regions. 

As mentioned before, the proposed type 3 structure consists of *N* number of Ag nanorods in the bus waveguide, enhancing the GPR effect between two adjacent Ag nanorods. As seen in Figure 8b, the λ_res_ has moved to the higher wavelengths without enlarging the device’s dimension. In other words, various resonance modes can be obtained by changing the metal defect’s numbers or their radii. Accordingly, this approach intends to decrease the footprints of the proposed type 3 structure, increasing with the number of Ag nanorods, thus making it easier for nanoparticles to excite plasmons in the presence of low restorative force in a positive-negative metallic background (i.e., dipolar effect). However, it has been found in Figure 8b that the variation in *N* number primarily affects the width of FWHM and does not have much effect on the shift in λ_res_ position. For example, in *N* = 21, λ_peak_ changes in mode 1 to mode 9 from 499 nm to 2025 nm, with the maximum transmittance fluctuating between 29.30% and 68.96%; λ_dip_ shifts from 523 nm to 2433 nm, with the minimum transmittance ranging from 0.063% to 3.10%. Hence, we adopted the stub height of *h* = 200 nm and metal defects’ *N* = 21 as the standard value for further studies.

**Figure 9:**
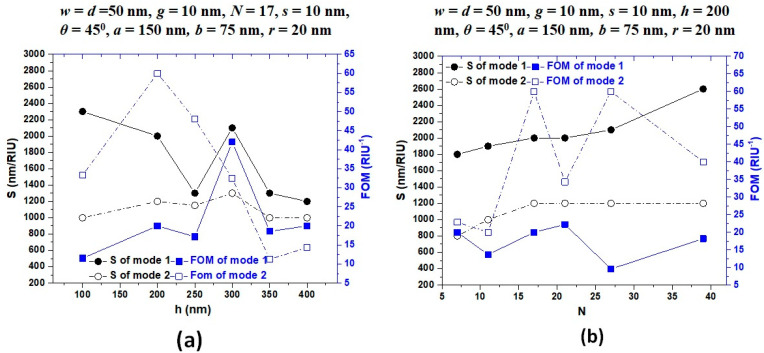
Sensitivity and figure of merit (FOM) of the proposed type 3 structure in mode 1 and mode 2 for varying (**a**) stubs height (*h*) in the range of *h* = [100, 200, 250, 300, 350, 400] nm, (**b**) varying the number of Ag nanorod in the bus waveguide (*N*) in the range of N = [7, 11, 17, 21, 27, 39] nm. The other structural parameters are shown on the top of the figures. The surrounding refractive index varies from 1.00 to 1.05 with an increment of 0.01.

Figure 9a,b shows the sensitivity (nm/RIU) and figure of merit (1/RIU) of mode 1 and mode 2 for the variation of *h* and *N* in type 3 structure. Results show that the sensitivity of all curves in mode 1 are higher than those in mode 2, while most of those FOM in mode 2 are higher than in mode 1. The calculated maximum sensitivity can reach 2600 nm/RIU and simultaneously achieve *S* = 2000.00 nm/RIU in mode 1 and 1200.00 nm/RIU in mode 2 when *h* = 200 nm and *N* ranging in 17 to 39, respectively. These values considerably raise the sensitivity by 216.67% for mode 1 and 133.33% for mode 2 compared to its counterpart without air stubs and Ag nanorods in type 2 structure. 

**Figure 10:**
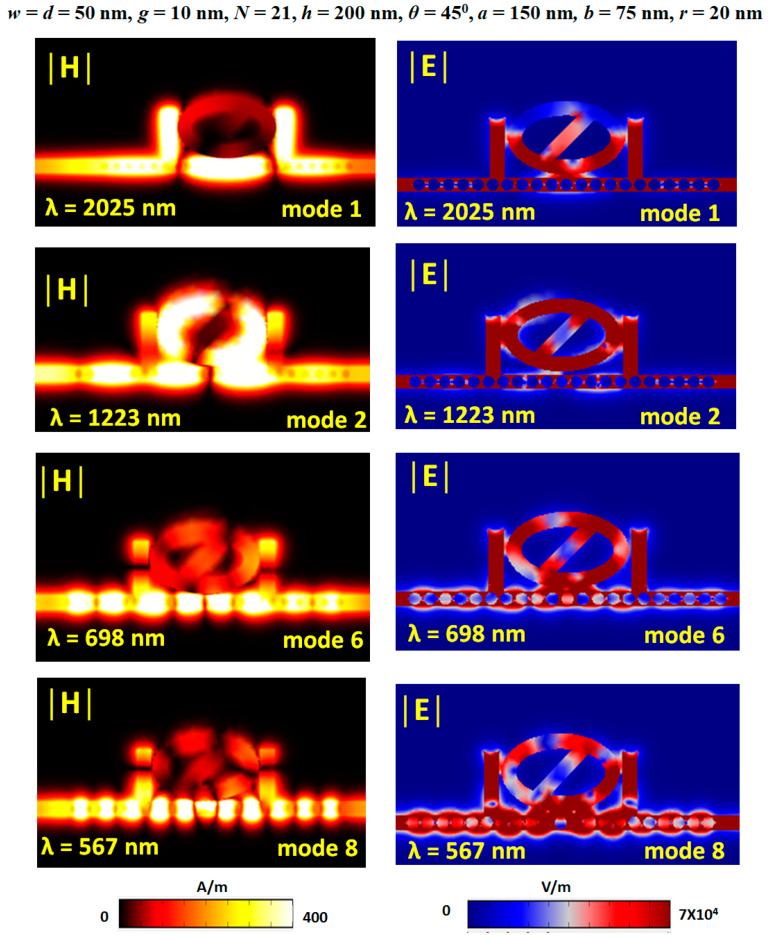
Selected plots of normalized (**a**) magnetic field intensity (|H|) and (**b**) electric field intensity (|E|) at corresponding resonance wavelengths of modes 1, 2, 6, and 8, respectively.

Figure 10 shows the selected plots of normalized magnetic field intensity (|H|) and electric field intensity (|E|) at corresponding resonance wavelengths of modes 1, 2, 6, and 8, respectively. The structural parameters used in these plots are at the top of this figure. In Figure 10, the different phases in the |H| field distributions can elucidate that the asymmetric Fano resonance line shape reveals in transmission spectra because the phase can significantly perturb in stubs, bus waveguide, and resonator. The |E| field distribution corresponding to the positive-negative charge distributions in forming attractive or restorative force occurred in a positive or negative metallic background. In addition, we observed that the position of light spots (i.e., high intensity of |E| field region) appears around the edges of the dual air stubs, air path, and Ag nanorods because the light spots’ position can affect the effective position coupling between the bus waveguide and resonator.

Furthermore, the air path orientation there upon affects the excitation of the resonance modes. The utilization of the split-ellipse resonator structure can give more flexibility in tailoring the transmittance properties. The desired working wavelengths and the number of resonance modes can be obtained by selecting the structural parameters of the proposed type 3 structures. These results imply that using dual stubs, air paths, and metal defects in the type 3 structure can lead to mode splitting of the Fano resonance.

**Figure 11:**
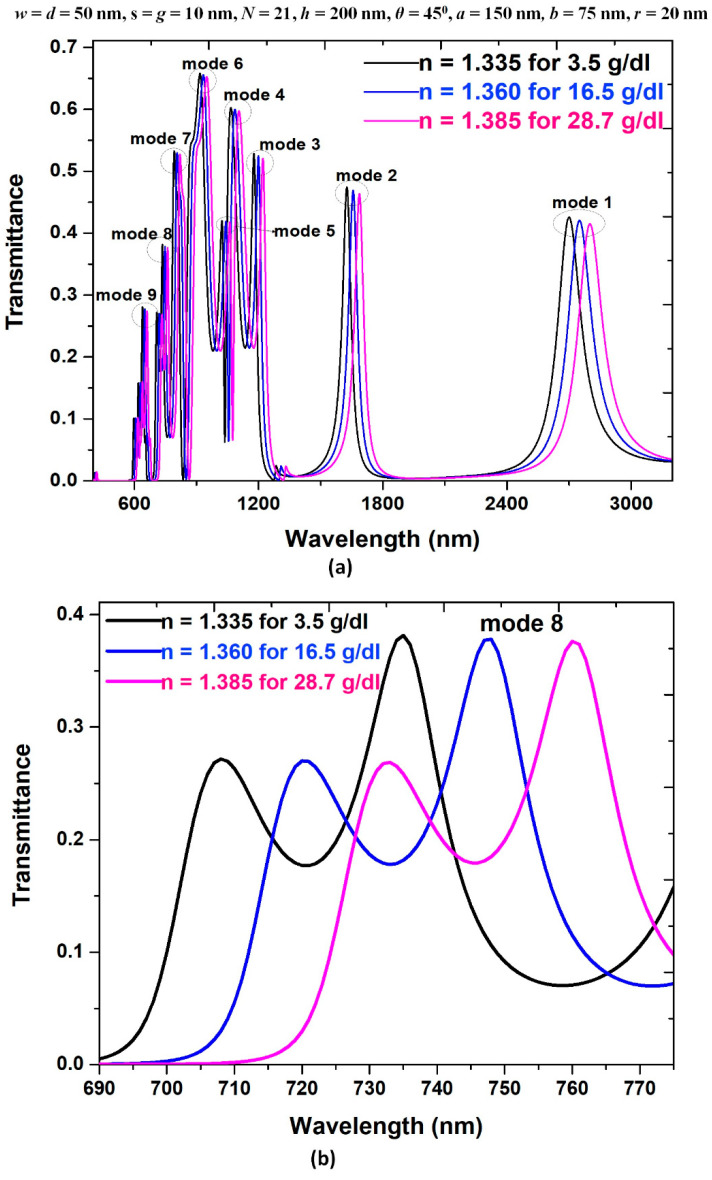
Transmittance spectrum of the proposed type 3 structure with three different concentrations of hemoglobin in a liquid (*n* = 1.335 for 3.5 g/dL, *n* = 1.36 for 16.5 g/dL, *n* = 1.385 for 28.7 g/dL) in the wavelength range of (**a**) 300–3200 nm for mode 1 to mode 9, and (**b**) 690–775 nm for mode 8, respectively. The structural parameters are on the top of this figure.

Figure 11a depicts the transmittance spectrum of the proposed type 3 structure with three different concentrations of hemoglobin in a liquid, i.e., *n* = 1.335 for 3.5 g/dL, *n* = 1.36 for 16.5 g/dL, *n* = 1.385 for 28.7 g/dL, see [79], in the wavelength range of 300–3200 nm from mode 1 to mode 9. We labeled the structural parameters on the top of this diagram. For applying the hemoglobin concentration of the human blood sample (see Section 5), we also plotted the transmittance spectrum of the proposed type 3 structure in the wavelength range of 690–775 nm for mode 8, as shown in Figure 11b. As seen in Figure 11a,b, the increasing refractive index would shift the transmittance peak wavelength to higher values, and the transmittance peaks can separate from one another. As a result, the sensitivity value of 2000 nm/RIU can achieve for the type 3 structure. The obtained results from the type 3 structure are remarked as higher than others reported literature. In Table 2, we summarized the sensitivity and FOM between this work and some other published articles. The sensitivity of our proposed device is higher, which shows the novelty of our design. Therefore, we can infer that the air path, dual air stubs, and metal defects in type 3 structure positively influence the coupling efficiency in the resonator. Based on the above studies and analyses, the sensing feature of the designed type 3 structure can be concluded as follows:

(1) The air path embedded in the elliptical-shaped resonator allows the mighty confinement of SPPs and offers destructive interference in the resonator. The resulting multiple Fano resonance modes in the type 3 structure can attribute to the resonator’s symmetry breaking, arising from the inherent resonant mode in the air path.
(2) The dual air stubs can function as a transformer for switching transmittance from dip to peak. The transmittance dip’s line shape can transfer to peak profile by adding the double air stubs connected to the bus waveguide and adjacent to the elliptical-shape resonator, contributing to the CPR and coupling effect between the stubs and resonator.
(3) The defect metals embedded in the bus waveguide can mediate the SPPs mode in the bus waveguide and serve as a buffer to mediate the GPR mode, significantly benefiting the SPPs modes in the proposed plasmonic-sensing system.
(4) Based on the linear relationship between the ambient medium’s refractive index and the transmittance dips/peaks of λ_res_, we can obtain the refractive index by detecting the dips/peaks’ λ_res_.

## 5. Application for Detection of Hemoglobin Concentration 

Hemoglobin concentration in human blood samples serves an imperative role in medical interventions in any sickness. Therefore, specific skills and highly sensitive sensors must accurately analyze the blood samples’ hemoglobin concentration. In real sensing applications, the hemoglobin concentration associates with the refractive index; consequently, the designed type 3 structure can detect the hemoglobin concentration. The blood sample can fill inside the air regions of stubs, elliptical-shape resonator, and bus waveguide. The plasmonic sensor demands a few slight drops of samples since the SPPs are very sensitive to the change of ambient materials. This result is very different from the conventional devices that require assembling blood samples of several milliliters [84]. In [79], Sharma et al. found that the sensitivity increases approximately 25% for any value of hemoglobin concentration when the wavelength increases from 700 nm to 1000 nm. Furthermore, Moritz Friebel et al. [33] determined the real part of the complex refractive index of native hemoglobin dependent on the concentration in the wavelength range of 250 to 1100 nm. They collected hemoglobin solutions directly from human erythrocytes, including other organic compounds and salts. The SPPs mode must happen at different λ_res_ because different blood groups have certain refractive indexes based on their specific chemical combinations. Li et al., experimentally studied the refractive index of blood groups (O, A, and B) at visible and near-infrared wavelengths (380–860 nm) for different blood samples and provided details of refractive index dispersion using the Cauchy formula. Therefore, we chose the working wavelengths of 250–1100 nm based on the above studies. 

The relationship between the blood samples’ refractive index and hemoglobin concentration can be described by the Cauchy formula [85].

*n*_*blood*_ = *n*_0_ + α*C* (6)

where *n*_0_ represents the effective refractive index of the blood sample when *C* is zero. *C* is the hemoglobin concentration of the blood sample (in g/L), and *α* is the specific refraction increment that fixes a particular blood group. Besides, the refractive index of human blood can also influence temperature T (in the Kelvin unit) and the working wavelength *λ* (nm). As a result, Equation (6) can be denoted by [86]:

*n*_*blood*_ = *n*_0_ + α*C* + β*T* + δλ + σλ^2^ +γλ^3^ (7)

where *α*, *β*, *δ*, *σ*, *γ* are Cauchy coefficients (constant values) that change with blood group. We show the importance of these coefficients in Table 3.

**Figure 12:**
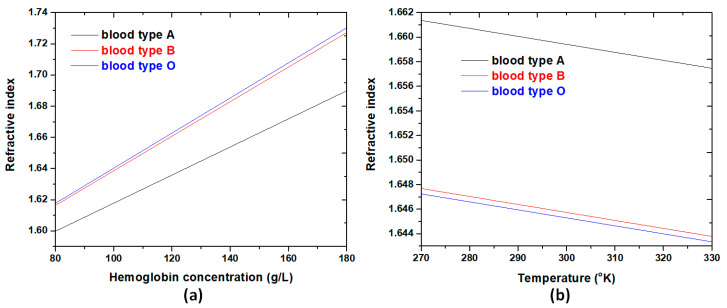
(**a**) Three blood types of refractive indices spectra versus hemoglobin concentration ranging from 90 g/L to 180 g/L for a selected working wavelength, λ = 735 nm, at T = 300 K. (**b**) Three blood types of refractive indices spectra versus the temperature in the range of 270–330 K with C_A_ = 132.5 g/L, C_B_ = 106.5 g/L, and C_O_ = 104.5 g/L for λ = 735 nm.

**Figure 13:**
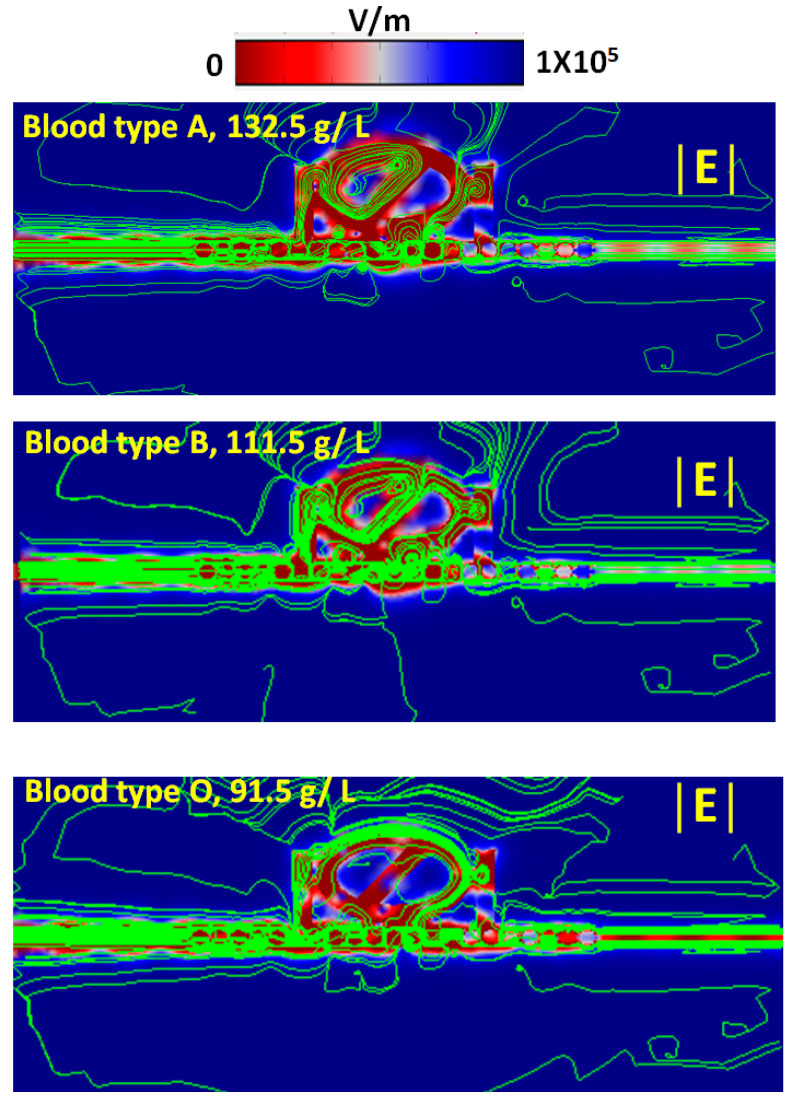
Selected normalized electric (|E|) field intensities including energy flows (Joul/m^2^) of three blood types of hemoglobin concentrations, i.e., 132.5 g/L, 111.5 g/L, and 91.5 g/L, for λ = 735 nm.

From Equation (3), λ_res_ is proportional to *n*_eff_ while the investigated structure’s effective size and mode number are fixed. As a result, one can expect that the excellent performance of the SPR sensor as shown in Figure 11 (refractive index of *n* ~ 1.3) is retained in other ranges of *n* (e.g., *n* > 1.60), and this result was verified by our previous literature (e.g., [87,88]). According to Equation (7), factors such as hemoglobin concentration and temperature are closely related to the refractive index variation of different blood groups. Figure 12a plots the refractive index variations corresponding to the three blood types within the hemoglobin concentration ranging from 90 g/L to 180 g/L for a selected working wavelength (λ = 735 nm) at temperature T = 300 K. As seen, the refractive index of the three blood types increases linearly with the increase of hemoglobin concentration. Consequently, we can obtain the related hemoglobin concentration if the refractive index can be achieved through the SPPs modes of inspection. Figure 12b also reveals the varying refractive index with temperature T in the range of 270–330 K at λ = 735 nm when the hemoglobin concentrations are selected as C_A_ = 132.5 g/L, C_B_ = 106.5 g/L, and C_O_ = 104.5 g/L, respectively. The obtained line shape also shows a linear growth trend. Going further deeper in understanding their mechanism, Figure 13 shows the selected normalized electric (|E|) field intensities, including energy flows (Joul/m^2^) of three blood types of hemoglobin concentrations (i.e., 132.5 g/L, 111.5 g/L, and 91.5 g/L). As observed, the |E| fields and energy flows well confine in the dual air stubs, air path, and metal’s gap regions. Significantly, the high-density energy flows appear in the interface between the double air stubs and resonators. Besides, the dense energy flows enclose the Ag nanorods and efficiently connect to the resonator, showing a better coupling effect between the bus waveguide and resonator. As the local environmental refractive index increases, the localized SPR peak will shift to a longer wavelength due to the reduced repulsion between dipoles with the same orientation, thus reducing the energy of the plasmon oscillations. It is important to note that the energy flows in each case formed a spherical-like spot between the bus waveguide and the direction of the air path with the rotation angle of *θ* = 45^0^. These results show that the measurement of hemoglobin concentration with the proposed type 3 structure can be conducted conveniently with high accuracy at the desired wavelength, and the proposed design can apply in diverse biomedical and refractive index-sensing applications.

## 6. Conclusions

This paper proposes a simple design strategy of a plasmonic sensor with multiple-mode Fano resonance for refractive index-sensing applications. We quantitatively used the finite element method to analyze the influence of the optical characteristics of transmittance spectra and sensor performance in three types of structures. The designed sensor has the advantage of easy fabrication and small volume and can flexibly tune the sensing characteristics because of the proposed plasmonic system’s dual air stubs, air path, and metal defects. The transmittance spectrum could dramatically change from dip to peak by adding the air stubs and metal defects. The simulation results show the multiple Fano resonance curves display in the transmittance spectrum, and the values of *s*, *r*, *h*, and *N* have different effects on the sensitivity and FOM. The physical features provide a highly efficient plasmonic sensor for refractive index sensing. The high sensitivity of 2600 nm/RIU and 1200 nm/RIU was simultaneously achieved in mode 1 and mode 2 of the proposed type 3 structure, which considerably raised the sensitivity by 216.67% for mode 1 and 133.36% for mode 2 compared to its counterpart without air stubs and Ag nanorods (i.e., type 2 structure). This research offers the theoretical foundation for comparing designed strategies for guided nanostructures that enable the measurement of a wide variety of refractive index medium and analytes, including gas, liquid, biomaterials (e.g., hemoglobin concentration), etc. We believe that the proposed structure can find significant applications in the future optical-sensing domain. 

## Figures and Tables

**Table 1:** Comparison of λ_res_, FWHM, ΔD and Q factor of type 1 and type 2 structures at resonance modes.

		Mode 1	Mode 2	Mode 3	Mode 4	Mode 5
Type 1	λ_res_ (nm)	1251	641			
	FWHM (nm)	70.00	25.00			
	ΔD (%)	91.21	78.83			
	Q-factor	17.87	25.64			
Type 2	λ_res_ (nm)	1189	949	774	627	492
	FWHM (nm)	15.00	28.00	10.00	15.00	10.00
	ΔD (%)	79.58	81.06	78.48	90.28	79.61
	Q-factor	22.12	33.89	77.40	41.80	49.20

**Table 2:** Comparison of the sensitivity and FOM between this work and some other published works.

Reference	Mode Number	Operating Wavelength Range	Max. Sensitivity (nm/RIU)	Max. FOM (1/RIU) or FOM*
[67]/2017	2	700 nm < λ_res_ < 1300 nm	840	FOM* = 100.00
[80]/2018	1	800 nm < λ_res_ < 2000 nm	880	FOM = 96.40
[81]/2019	2	700 nm < λ_res_ < 1600 nm	760	FOM* = 9.9 × 10^4^
[82]/2020	2	700 nm < λ_res_ < 2000 nm	2300	FOM = 31.50
[83]/2021	4	400 nm < λ_res_ < 1300 nm	932	FOM = 710.00
This work	8	400 nm < λ_res_ < 1500 nm	2600	FOM = 60.00

**Table 3:** Model coefficients used in Equation.

Parameter	Unit	A-Group	B-Group	O-Group
n_0_	--	1.54712	1.54712	1.54712
α	L/g	9.014 × 10^−4^	11.09 × 10^−4^	11.26 × 10^−4^
β	1/°K	−6.497 × 10^−5^	−6.497 × 10^−5^	−6.497 × 10^−5^
δ	nm^−1^	−8.47 × 10^−6^	−8.47 × 10^−6^	−8.47 × 10^−6^
σ	nm^−2^	7.08 × 10^−7^	8.014 × 10^−7^	7.742 × 10^−7^
γ	nm^−3^	−1.28 × 10^−10^	−2.286 × 10^−10^	−1.823 × 10^−10^

## Data Availability

Not applicable.
